# Circadian clock genes, ovarian development and diapause

**DOI:** 10.1186/1741-7007-8-115

**Published:** 2010-09-03

**Authors:** William E Bradshaw, Christina M Holzapfel

**Affiliations:** 1 Center for Ecology and Evolutionary Biology, University of Oregon, Eugene, OR 97403-5289, USA

## Abstract

Insects, like most organisms, have an internal circadian clock that oscillates with a daily rhythmicity, and a timing mechanism that mediates seasonal events, including diapause. In research published in BMC Biology, Ikeno *et al. *show that downregulation of the circadian clock genes *period *and *cycle *affects expression of ovarian diapause in the insect *Riptortus pedestris*. They interpret these important results as support for Erwin Bünning's (1936) hypothesis that the circadian clock constitutes the basis of photoperiodism. However, their observations could also be the result of pleiotropic effects of the individual clock genes.

See research article http://www.biomedcentral.com/1741-7007/8/116

## 

There are two major rhythms of the biosphere, a daily cycle of night and day, and an annual seasonal cycle marked by changes in day and night length. Temporal coordination with both these cycles is important for the maintenance of fitness in animals [1]: the daily cycle is tracked by an internal circadian clock that governs a large array of daily biochemical and physiological responses, while the seasonal cycle stimulates photoperiodic responses that can be crucial to survival, as in the case of insect dormancy, or diapause, which shuts down reproduction and reduces metabolic needs in response to shortening days. In 1936, Erwin Bünning [2] proposed that circadian rhythmicity constituted the basis of photoperiodic time measurement, a hypothesis with intrinsic appeal given that both processes rely primarily on the input of light. However, the hypothesis remains far from proven. Many studies at the physiological level have claimed to support a circadian involvement in photoperiodism, but others have challenged the connection. At a molecular level, much is now known about the details of the circadian clock in *Drosophila *and other insects [3,4] but there has been no clear demonstration that the circadian clock, as a functional module, underpins photoperiodism, whose molecular basis remains unknown. There is, however, evidence that the circadian clock gene *timeless *influences diapause in the dipteran *Drosophila *[5]. Now, Ikeno *et al. *[6] report experiments using RNA interference (RNAi) to target two core clock genes in the heteropteran bean bug *Riptortus pedestris*, showing that inhibition of either *cycle *or *period *expression disrupts a circadian rhythm of cuticle deposition, and at the same time affects ovarian diapause. For reasons we shall explain more fully below, they interpret these important results as support for Bünning's hypothesis, while we argue that pleiotropic effects of the *cycle *gene are likely at play.

## Simplified circadian clockworks

**Figure 1:**
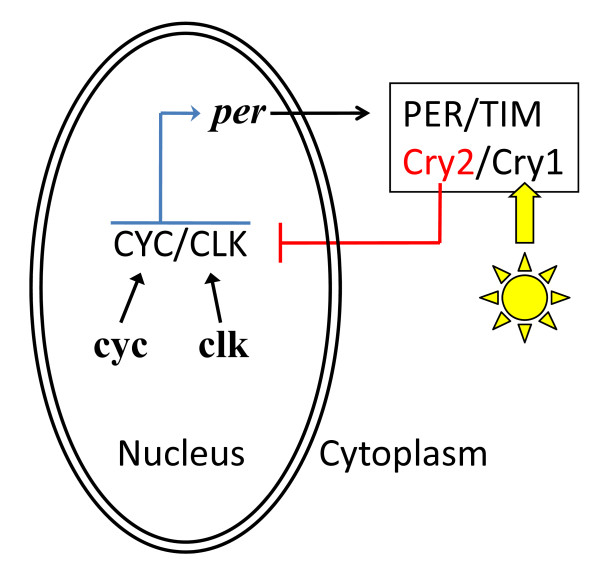
**Simplified schematic of circadian clockworks**. This schematic is based on the likely state of ancestral insect clocks [3,4] and includes the two genes manipulated by Ikeno *et al. *[6]. The transcription of *cycle *(*cyc*) is continuous and that of *clock *(*clk*) is rhythmic. The translated proteins (CYC and CLK) form a CYC-CLK heterodimer that promotes the transcription of *period *(*per*). *per *mRNA is transported to the cytoplasm where it is translated into its protein (PER) and forms a complex with the *timeless *protein (TIM), and two paralogous proteins of cryptochrome (CRY1 and CRY2). CRY1 is the likely photoreceptor that enables the circadian clock to entrain to daily light:dark cycles. CRY2 is a light-insensitive protein that acts as the main transcriptional repressor of the circadian clockworks. In *Riptortus pedestris *[6], double-stranded RNA directed against *cyc *(*cyc *RNAi) effectively reduces *cyc *and, as expected, *per *expression; *per *RNAi effectively reduces *per *expression and would not be expected to reduce *cyc *expression. Hence, the principal difference between the effects of *cyc *and *per *RNAi at the molecular level would be the level of *cyc *expression, since *per *expression is blocked in both cases. Also, the reduced levels of *cyc *and *per *that they achieve result in arrhythmicity of daily cuticle deposition, that is, render the circadian clock dysfunctional.

The basic circadian clock of insects functions as a light-sensitive molecular oscillator, incorporating a light-sensitive protein known as CRYPTOCHROME (CRY) and various feedback loops with positively and negatively acting elements. Common to all insect circadian clocks (Figure 1) is the transcription and translation of the genes *cycle *and *clock*, whose protein products form a heterodimer (CYC-CLK) that promotes transcription of *period *(*per*). In the cytoplasm, the protein encoded by *per *(PER) interacts with a number of other clock proteins that include TIM (encoded by *timeless*) and CRY (encoded by *cryptochrome*). Negative feedback of CYC/CLK activity appears to build through the night but is relieved at dawn when light triggers the degradation of TIM. Due to ancestral gene duplication, there may be two *cryptochrome *genes in any given insect species - *cry1 *and *cry2 *(*cry2 *is called *cry-m *in *R. pedestris*). The ancestral circadian clock probably involved CYC as the positive-acting transcriptional activator, CRY1 as the main photoreceptor, and CRY2 as the light-insensitive, negative-acting transcriptional regulator. In *Drosophila*, PER is the main negative regulator of clock function, but so far, in all insects where *cry2 *is present and regardless of *cry1*'s presence, CRY2 plays this role [4]. Thus, in *R. pedestris*, which has *cry2 *[7], *cyc *likely acts as the main positive transcriptional activator and *cry2 *as the main negative regulator, while *per *is likely involved in the negatively acting portion of the feedback loop.

## The effects of breaking the clock in *R. pedestris*

Ikeno *et al. *suppressed the expression of the clock genes *cycle *and *period *by injecting bean bugs with double-stranded RNA (RNAi), showing by northerns that this achieved at least partial downregulation of the targeted gene, and also a reduction in *per *mRNA after *cyc *RNAi treatment, which would be expected as a secondary consequence of reduced levels of CYC (Figure 1 in [6]). As a read-out for the function of the circadian clock they looked at the layering of the cuticle, which appears as alternating bright and dark layers under polarized light, and is laid down in a rhythm they established as showing the classic features of being regulated by a circadian clock (Figure 1 in [6]). Injection of *cyc *RNAi resulted in the loss of alternate layering and instead the deposition of a single bright layer. Injection of *per *RNAi also resulted in the loss of alternate layering, but the outcome was a single dark layer (Figure 3 in [6]). Thus, in both cases there was a loss of circadian rhythm, but with a different phenotypic consequence, and the authors interpret this as an arrest of the clock in two different phases, leading to the activation of distinct downstream cascades.

**Figure 2:**
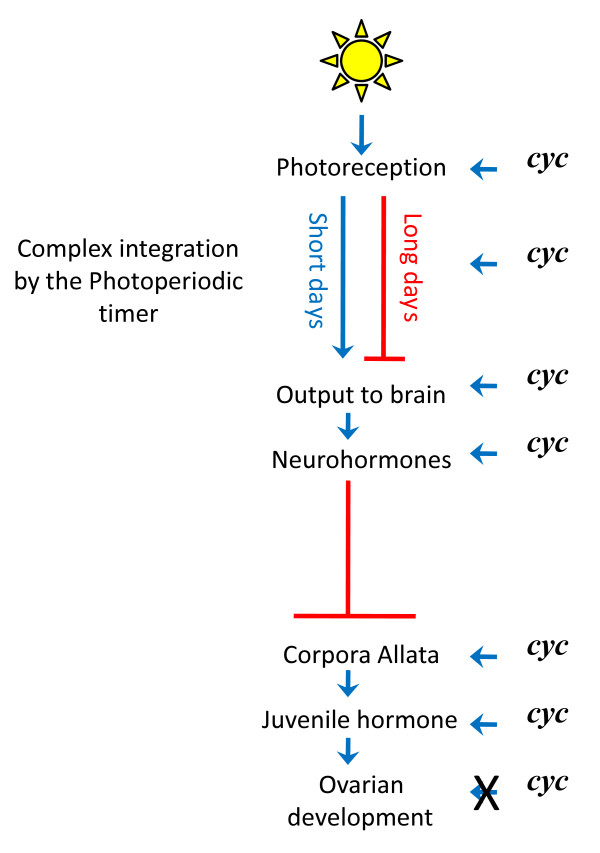
**Simplified sequence of events from light to diapause in *R. pedestris***. Light stimulates photoreceptors [9] whose output is integrated by an unknown, complex set of events within the photoperiodic timer and is translated into a long- or short-day signal to the brain. Long days inhibit and short days stimulate the production of neurohomones by neurosecretory cells in the brain [8]. The neurohormones produced by these cells inhibit the corpora allata from secreting juvenile hormone, a terpenoid that is necessary to promote ovarian development. Hence, long days promote ovarian development by blocking inhibition of the corpora allata while short days promote diapause (non-development) by promoting inhibition of the corpora allata. Ikeno *et al. *[6] show that *cycle *(*cyc*) is acting at the level of juvenile hormone secretion by the corpora allata, or at one of many unknown, higher levels in the complex cascade of events initiated by light.

The remarkable result was that *per *and *cyc *RNAi also affected ovarian diapause. Normally *R. pedestris *undergoes an ovarian diapause that is controlled by the length of day (Figure 2). Long days promote ovarian maturation by inhibiting neurohormones from the brain that prevent synthesis or secretion of juvenile hormone from the corpora allata [8]. Short days permit expression of the inhibitory neurohormones and, ultimately, result in non-developing ovaries (diapause). When there were significant differences from controls, *per *RNAi increased the incidence of ovarian development and *cyc *RNAi decreased the incidence of ovarian development (Figure 4 in [6]). Consistent with these results, and indicating an action on the regulatory cascade leading to diapause at the level of juvenile hormone or upstream, *per *and *cyc *RNAi had opposite effects on expression of genes known to be up- or down-regulated by juvenile hormone (Figure 5 in [6]). Furthermore, the application of a juvenile hormone analog (methoprene) restored ovarian development in *cyc *RNAi bugs (Figure 6 in [6]). Thus, *cyc *is involved in regulating diapause somewhere between the input of light and the secretion of juvenile hormone in the corpora allata (Figure 2).

## Support for Bünning's hypothesis or pleiotropic effects of *cyc*?

Ikeno *et al. *see parallels in their results that lead them to claim support for Bünning's hypothesis. They suggest that having targeted the principal positive regulator of the bean bug's clock (*cyc*) and a negative regulator (*per*), they have stopped the circadian clock at different phases of its oscillating cycle. It is unable to oscillate in response to the night-day cycle, but having become stuck in opposing phases, output signals are still delivered to give phenotypes that correspond, in the case of the cuticle, to those normally associated with opposite swings of the night-day pendulum. In the case of diapause, the differences in sensitivity to day length are interpreted as disruption of the photoperiodic timer, with the switch for diapause stuck in either one of two opposing positions. Thus, they see support for Bünning's proposition that the circadian clock mechanism lies at the heart of photoperiodicity.

These are, however, speculative suggestions only, venturing beyond the data, and in our view biased by the intrinsic appeal of connecting the circadian clock and photoperiodic time measurement. Based on the data, we would make the following points. First, both RNAi treatments lead to a reduction of *per *expression, so that any phenotypic differences observed must be attributed largely to the difference in *cyc *expression. Second, both treatments rendered the circadian clock dysfunctional. Third, all of the phenotypes that varied between the two RNAi treatments did so in the consistent absence of a functional circadian clock. If there is consistently no functional circadian clock in both RNAi treatments, then phenotypic differences between those treatments cannot be ascribed to the circadian clock. We would therefore conclude that the circadian clock as a functional unit (module) does not provide the essential clockworks for photoperiodic time measurement in *R. pedestris*.

Just because the circadian clock is dysfunctional does not mean that individual clock genes have no other pleiotropic effects [9]. The diapause response of bugs exposed to long and short days (Figure 4 in [6]) can be considered in terms of the presence of *cyc *expression (*per *RNAi treatment) or absence of *cyc *expression (*cyc *RNAi treatment). When *cyc *is expressed, there is an increase in ovarian development (non-diapause) compared to when *cyc *is not expressed, regardless of day length. The application of a juvenile hormone analog tells us that *cyc *is not acting at the level of the ovaries themselves. However, these results do not tell us where *cyc *is exerting its effect. This could be anywhere in the cascade of events (Figure 2), from the input of light [10] to the secretion of juvenile hormone in the corpora allata. Given that *cyc *encodes a positive transcriptional regulator, multiple pleiotropic effects are possible, and even likely.

## Progress in the understanding of diapause

In sum, Bünning's hypothesis remains in contention but unproven in insects. Nonetheless, Ikeno *et al. *[6] have made significant advances in the understanding of the expression of diapause. They have shown in *R. pedestris *that the circadian clock gene *cyc *plays an important role in ovarian development or diapause, and that it is involved somewhere in the upstream part of the pathway of juvenile hormone and not at the level of the ovaries themselves (Figure 2). Finally, they have added *cycle *in Heteroptera to *timeless *in Diptera [5] as core circadian clock genes that also have independent effects - on insect diapause. Their research establishes *cyc *as an important and interesting focus for future research.

## Abbreviations

*cry*: *cryptochrome *(a circadian clock gene); *cry1*: a *Drosophila*-like duplicate of *cry*; *cry2*: a vertebrate-like duplicate of *cry*; *cyc*: *cycle *(a circadian clock gene); *per*: *period *(a circadian clock gene); RNAi: RNA interference; *tim*: *timeless *(a circadian clock gene).
